# *In vitro* Prebiotic Properties of Garlic Polysaccharides and Its Oligosaccharide Mixtures Obtained by Acid Hydrolysis

**DOI:** 10.3389/fnut.2021.798450

**Published:** 2021-12-09

**Authors:** Xiaoming Lu, Ningyang Li, Renjie Zhao, Meng Zhao, Xuanxuan Cui, Yukun Xu, Xuguang Qiao

**Affiliations:** Key Laboratory of Food Processing Technology and Quality Control in Shandong Province, College of Food Science and Engineering, Shandong Agricultural University, Tai'an, China

**Keywords:** garlic, polysaccharide, oligofructose, acid hydrolysis, prebiotic effect

## Abstract

Fructans and oligofructose are usually used as prebiotics without any limitation in functional food or food ingredients. The degree of polymerization (DP) of polysaccharides affects the utilization of probiotics. Garlic is rich in fructans. The objective of this study was to extract and purify polysaccharides from garlic, analyze its composition, hydrolyze them using HCl, and then evaluate the prebiotic potential of the garlic neutral polysaccharides (GPs) before and after hydrolysis. GPs were 6.57 × 10^3^ Da with a composition of fructose and glucose at a ratio of 4:1. After acid hydrolysis, low molecular weight fraction in garlic oligofructose (GOs) may be eliminated through ultrafiltration. The content of oligosaccharides with an average DP < 10 increased from 15 to 75%. GPs and GOS had a stronger resistance to acid conditions in human stomach than fructooligosaccharide, and GOs showed better prebiotic properties on the growth of lactobacilli than GPs. This study evaluates the prebiotic potential of the garlic frutctans and oligosaccharides mixtures obtained by acid hydrolysis, which may be used as an ingredient in functional food and nutraceutical products.

## Introduction

Garlic (*Allium sativum* L.), a species of the onion family *Alliaceae*, has been used worldwide as a spice and traditional medicine for several centuries. It has been confirmed to have numerous favorable biological and pharmacological benefits such as anticancer, antioxidant effects, antimicrobial properties, and in preventing many diseases (1–3). Many of the biological effects are mainly due to the presence of organosulphur-containing compounds, although the major component of garlic is fructans, accounting for 70–80% of dry matter. They have been confirmed to be inulin-type and belong to the neokestose family, which has a (2→1)-linked β-D-Fru*f* backbone with a (2→6)-linked β-D-Fru*f* side chains. A high molecular weight fructan was isolated from raw garlic extract and the degree of polymerization (DP) was confirmed at about 58 (4). In addition, a series of oligofructose and fructans were extracted by water and ethanol, with molecular weight (*M*_W_) found in the range of 1,000–4,500 Da with a ratio of fructose: glucose = 15:1, corresponding to DP as high as 38 (5).

Studies on prebiotics and probiotics attract much attention from scientists and researchers recently. Prebiotics are non-digestible food ingredients that can beneficially affect the consumers' health by stimulating the selective growth of a limited number of bacteria in the large intestine, thereby improving the consumer's health (6). Fructans and oligofructose are usually used as prebiotics in functional food or food ingredients, which are used without any limitation. They represent a low caloric sweetener and have similar effects to those of dietary fiber. They can resist hydrolysis and digestion in the human upper gastrointestinal intestinal tract (7). This attribute constitutes them to be quantitatively fermented by competitive microflora in the colon, such as bifidobacteria and lactobacilli, which generate adverse conditions for Salmonella colonization (8, 9). Lactobacillus is known as one of the most beneficial probiotics to human health amongst more than 400 species of bacteria in the adults' intestine (10). They can produce carbohydrate-degrading enzymes to ferment the oligosaccharides to produce short chain fatty acids, resulting in significant changes in the composition of gut microflora by increasing the number of potentially health-promoting bacteria and reducing the number of potentially harmful species (11).

The degree of polymerization of polysaccharides affects the utilization of probiotics. Different inulin-type fructans have probably different efficacies (in terms of effective daily dose), and the most active product is oligofructose-enriched inulin (12). These results indicate that highly polymerized fructans are not efficiently utilized by bifidobacteria (13). Li et al. found that the optical density (OD) of culture medium was larger with lower DP of inulin as carbon source, which meant that the inulin with lower DP had a better prebiotic effect (14). Huang and Chen obtained the mixture of oligofructose and fructans with different DP through different concentrations of ethanol and found that the lactic fermentability increased, whereas the DP declined (15). Acid-hydrolysis is commonly used in polysaccharides degradation. It can degrade polysaccharides from high DP to low DP. It has been studied that the chain scission of the glycosidic linkage of polysaccharides during acid degradation appears to follow a first-order reaction (16, 17). Fructans with different molecular sizes can be degraded to glucose, fructose, sucrose, and low *M*_W_ fructo-oligosaccharides (18).

The physicochemical property and the biological activity of fructans may be different due to their different Mw; thus it appeared very interesting to isolate fructans from garlic, degrade them to low molecular saccharides by acid-hydrolysis, and then evaluate the prebiotic properties of them *in vitro*. Additionally, the prebiotic properties were elucidated in supporting the growth of L. *plantarum*, L. *john*sonii, L. *paracasei*, and L. *casei*. It may have a potential of garlic fructans and oligofructose as a source of prebiotic food ingredient for food applications.

## Materials and Methods

### Materials

Garlic without disease, insect injury, and mechanical damage was purchased from Jinxiang (Shandong, China) and then stored in dry and dark depots.

Calcium chloride, hydrochloric acid, phenol, sodium azide, sulfuric acid, trifluoroacetic acid, sodium nitrate, and 3,5-dinitrosalicylic acid were analytical grade and supplied by Sinopharm Chemical Reagent Co. Ltd (Beijing, China). Monosaccharide standard substances (Arabinose, mannose, galactose, glucose, and fructose) were purchased from Aladdin Chemical Reagent Co. Ltd (Shanghai, China). Dextran standard substances (Mw from 505 Da to 62 kDa) were purchased from Shodex. Deionized water (18.2 M resistivity) was obtained from a Milli-Q Element water purification system (Millipore, Bedford, MA). UV–Vis absorption spectra were recorded on a UV-Vis spectrophotometer (T6, Purkinje General, China). All the other chemicals used were of analytical grade.

### Extraction of the Garlic Neutral Polysaccharides

Garlic cloves were boiled at 100°C for 10 min to inhibit the activity of enzymes and then mixed with water in the ratio of 1:6, homogenized, and extracted for 2 h at 70°C. Six layers of cotton cheese cloth were used for filtration. To remove garlic pectin, the obtained filtrate was adjusted to pH 8.5 with ammonium hydroxide, and 10% (m/v) CaCl_2_ was added until no precipitate was generated. The mixture was centrifuged at 5,000 rpm for 10 min. Then the supernatant was concentrated to appropriate amount using a rotary evaporator at 60°C. The protein was removed by Sevag reagent (4:1, v/v) six times (19, 20). After the Sevag treatment, crude **garlic neutral polysaccharides** (GPs) solution was precipitated with a final concentration of 95% ethanol. The precipitate was collected and lyophilized. The crude GPs were purified by a column (2.6 cm × 60 cm) of DEAE-52 cellulose. The column was eluted by water at a flow rate of 0.5 ml/min and the obtained elute (5 ml/tube) was collected automatically and the polysaccharides were detected by the phenol–sulfuric acid method. The fraction of GPs were collected, concentrated, and lyophilized. On GPs were performed full-wavelength scanning in the range of 200–900 nm by a UV-2450 spectrophotometer (Shimadzu, Japan).

### Fourier Transform Infrared Spectroscopy

**Fourier transform infrared** (FTIR) spectra of GPs were directly measured using Nexus 670 FTIR spectrum analyzer (Thermo Scientific, USA). Spectral region ranged from 4,000 to 600 cm^−1^ with a resolution 4 cm^−1^ and 128 scans. Samples were prepared by grinding 1 mg of GPs with 150 mg of KBr and pressing the mixture into very thin disks.

### Analysis of Monosaccharide Composition

The hydrolysis process was applied to monosaccharide composition analysis (21). Trifluoroacetic acid (TFA) (2 M, 1 ml) was added to 20 mg of GPs sample. The mixture was incubated at 100°C for 5 h. Excess acid was removed by repeated evaporation of the hydrolyzate with methanol until dryness. The obtained mixture of monosaccharides was dissolved in triple-distilled water. The monosaccharides composition was analyzed by HPLC with a Zorbax carbohydrate column (250 mm × 4.6 mm, 5 μm). Mobile phase was acetonitrile: water (80:20) and the flow rate was 1.0 mL/min. Refractive index (RI) detector was used and the column oven temperature was 35°C. Arabinose, mannose, galactose, glucose, and fructose were used as monosaccharide standards. Qualitative and quantitative analysis of monosaccharides in the hydrolyzate was determined by comparison with the retention time and standard curve of sugars.

### Determination of *M*_W_ Distribution of Saccharides

The *M*_w_ distribution of carbohydrate was measured by gel permeation chromatography (GPC) on an LC-20AT HPLC system (Shimadzu, Kyoto, Japan) with a RID-10A refractive index detector. Samples were separately dissolved in triple-distilled water (10 mg/ml). The column employed was Shodex OHpak SB-803HQ, SB-802.5HQ, and SB-802HQ (300 mm × 8 mm, 6 μm) connected in series. The determination conditions were as following: mobile phase (0.1 M NaNO_3_ and 200 ppm NaN_3_; flow rate 0.3 ml/min), column temperature (35°C), injection volume (20 μl). The *M*_w_ was determined using the calibration curve given by the following equation:


$$\begin{array}{l} \text{Y}=-5.3967\times 10^{-5}{\text{X}}^{3}+1.3438\times 10^{-2}{\text{X}}^{2}-1.1623\text{X}+\\ 37.4904\;\left(R^{2}=0.9998\right) \end{array}$$

where *Y* is the *M*_w_ of polysaccharides samples, and *X* is the peak elution time of polysaccharides samples.

### Hydrolysis of GPs Mediated by HCl

The GPs were dissolved in distilled water to give a 10% (w/v) solution and then the solution was acidified by 1 M HCl to reach a pH 3. Hydrolysis reaction of the solution was carried out at 50°C. The solution was taken periodically for ultrafiltration by 3-kDa membrane and then repeated hydrolysis as above was performed to obtain oligofructose (22).

### Purification Method

In order to obtain garlic oligofructose (GOs), the hydrolyzate was subjected to ultrafiltration using 300 Da membrane to remove the reducing sugars (mostly fructose and some glucose). The retentate was concentrated by a rotary evaporator (Eyela, Tokyo, Japan) at 60°C and lyophilized by a freezing dryer (Scientz, Ningbo, China).

### Effects of Artificial Human Gastric Juice Hydrolysis

The non-digestibility of saccharides can resist digestive processes by gastric acidity and gastrointestinal absorption. Digestibility of GPs and GOs was tested by calculating the degree of its hydrolysis by artificial human gastric juice. Artificial human gastric juice was mimicked by using hydrochloric acid buffer containing (in g/L) : NaCl, 8; KCl, 0.2; Na_2_HPO_4_·2H_2_O, 8.25; NaHPO4, 14.35; CaCl_2_·2H_2_O, 0.1; MgCl_2_·6H_2_O, 0.18. The buffer was adjusted to pH 1, 2, 3, 4 and 5 using 5 M HCl (23). Sample solution (1% w/v, 5 ml) was added to the same volume of the buffer and incubated in the water bath (37 ± 1) °C for 6 h. The mixture was taken periodically at 0, 0.5, 1, 2, 4, 6 h and tested for reducing sugar content using DNS method (20). The total sugar content was determined by phenol–sulfuric acid method (24). Percentage of hydrolysis of saccharides was calculated based on the reducing sugar released and total sugar content of the sample as below:


$$\begin{array}{l} \%\text{ Hydrolysis}=\\ \frac{\text{reducing sugar released}}{\text{total sugar content }-\text{ initial reducing sugar content}}\times 100 \end{array}$$

where reducing sugar released is the difference between its final and initial content.

### Prebiotic Effect on Lactobacillus

The prebiotic effect of GPs and GOs was evaluated through the L. *plantarum*, L. *johnsonii*, L. *paracasei*, and L. *casei* growth. Carbohydrate-free Man-Rogosa-Sharp (MRS) medium was used as a basal growth medium. To examine the effect of different saccharides on prebiotic effect, the bacteria were cultivated in the basal medium containing glucose, sucrose, fructooligosaccharide (FOS), GPs and GOs, and the medium with no carbon was as control. Microbial growth was determined by OD measurement. The samples were measured spectrophotometrically using a UV–Visible spectrophotometer at 600 nm. Results were expressed as unit of cell densities. The pH of fermented broth over time interval was determined using a pH meter (Mettler Toledo, USA), which was calibrated using buffers of pH 4.0, pH 6.86, and pH 9.18 prior to analysis. The prebiotic index (PI) equation is described below:


$$\begin{array}{l} \text{PI}=\frac{{\text{A}}_{\text{PP}48}-{\text{A}}_{\text{PP}0}-\left({\text{A}}_{\text{PN}48}{-}_{\text{PN}0}\right)\;}{{\text{A}}_{\text{PG}48}-{\text{A}}_{\text{PG}0}-\left({\text{A}}_{\text{PN}48}-{\text{A}}_{\text{PN}0}\right)\;} \end{array}$$

*A*_PP0_ and *A*_PP48_ denote the OD value of the culture containing saccharides except glucose as carbon source at 0 and 48 h; *A*_PG0_ and *A*_PG48_, the OD value of the culture with glucose as carbon source at 0 and 48 h; *A*_PN0_ and *A*_PN48_, the OD value of the culture with no carbon source at 0 and 48h.

### Statistical Analysis

Data were analyzed by using one-way analyses of variance (ANOVA). Significant difference among treatments was calculated using Duncan's multiple range test at *p* < *0.05*. The statistical analysis of data was performed using SPSS software version 18.0 (IBM Corporation, Chicago, IL, USA).

## Results and Discussion

### *M*_W_ Distribution and Monosaccharide Analysis

**Figure 1:**
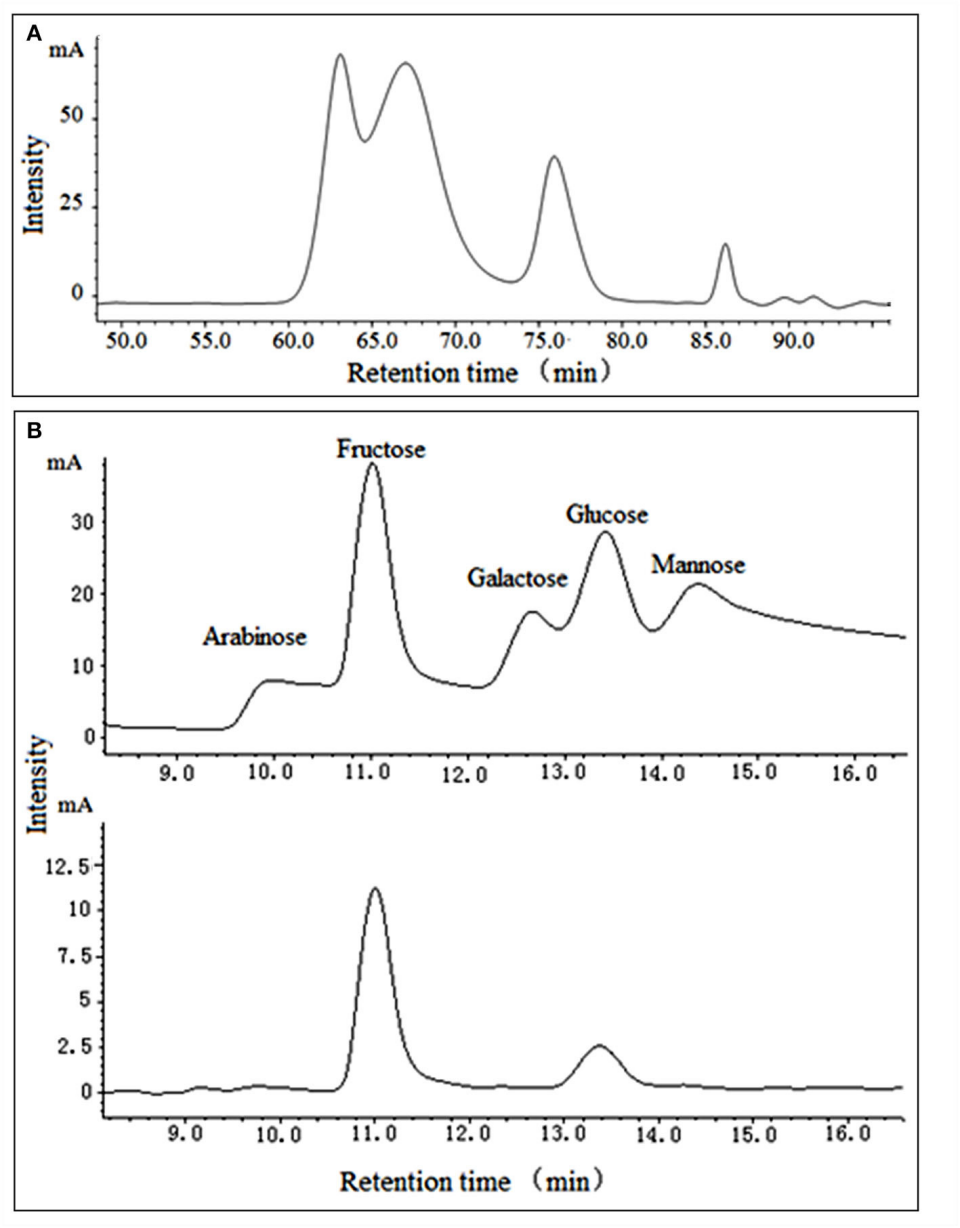
*M*_w_ distribution **(A)** and monosaccharide analysis **(B)** of purified GPs.

As garlic fructans are a polydisperse mixture of oligomers with varying DPs, GPs were characterized by their average *M*_W_ distribution. GPs, which showed no absorption at 280 nm in the UV spectrum, indicated the absence of protein and appeared as a white powder. The chromatogram (Figure 1A) showed that it was composed of saccharides with different M_W_, indicating that it was a polydisperse mixture of oligomers with varying DPs. Its average *M*_W_ was estimated to be 6.57 × 10^3^ Da by GPC, based on the calibration curve established using standard dextrans, which were assigned as previously described (25).

The monosaccharide composition of GPs was determined by TFA hydrolysis and followed by HPLC analysis (Figure 1B). It was mainly composed of fructose and glucose at a ratio of 4:1, which indicated that the predominant monosaccharide of GPs we obtained was fructose. Chen et al. revealed that garlic fructans were composed of fructose and glucose at a ratio of 14:1 (26). Jack and Shuryo extracted oligofructose and fructans and then analyzed their composition as fructose: glucose at 15:1 (5). The differences got by the independent researchers may be due to the different production processes or materials. Moreover, under high temperatures and acid condition, the fructose is easily dehydrated and oxidized to other products, such as glucose or 5-HMF, resulting in the difference between the measurement and actual value (27).

### FTIR Analysis of GPs

**Figure 2:**
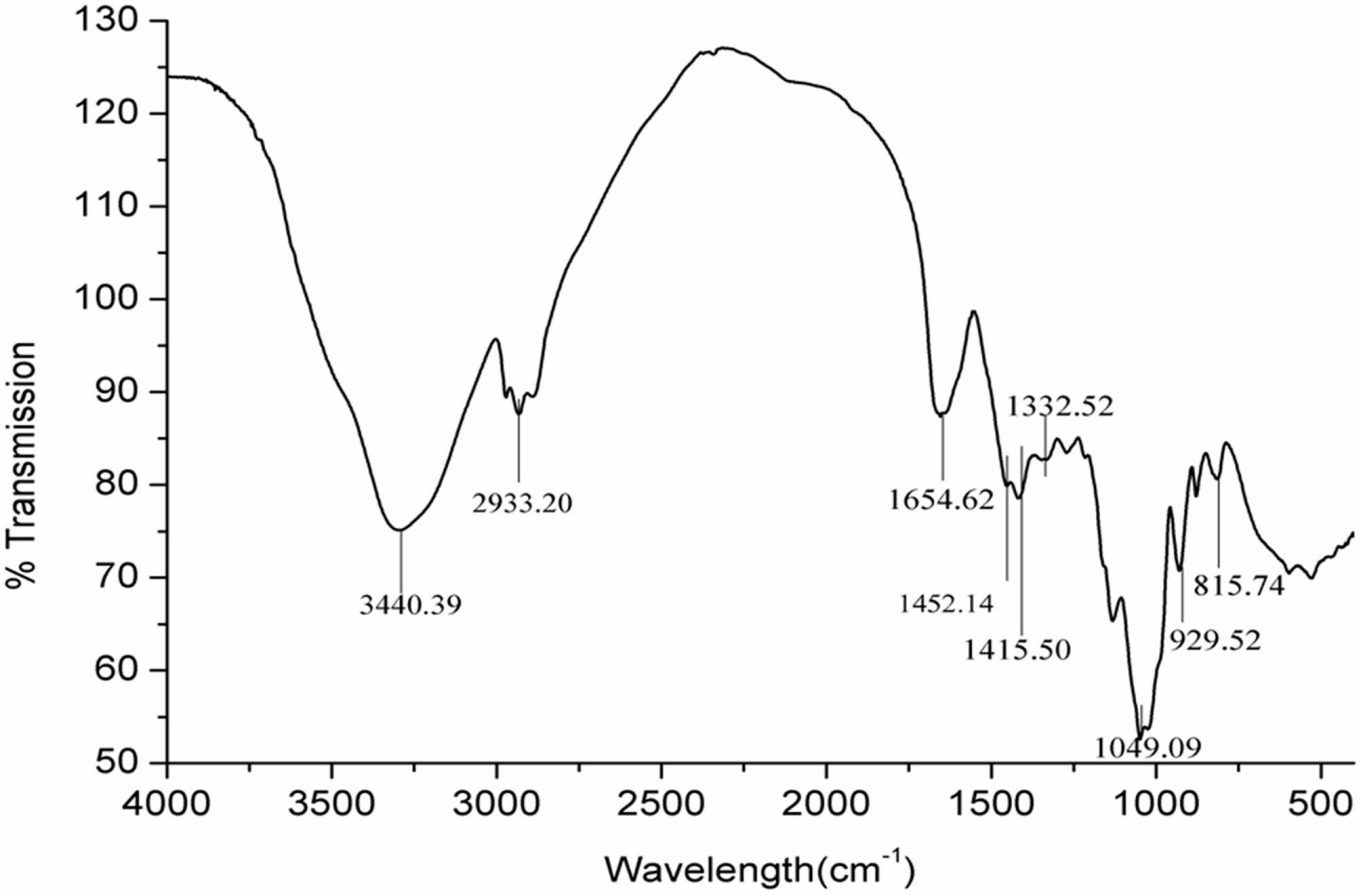
FTIR spectra of the GPs from garlic.

**Fourier transform infrared** spectroscopy can be used for the approximate identification of compounds when combined with data from chemical analysis. Types of monosaccharide, glycosidic bonds, and functional groups can also be determined using FTIR spectroscopy (28). A broad band centered at 3,440.39 cm^−1^ was assigned to OH, whereas the band centered at 2,933.20 cm^−1^ was CH stretching as a characteristic of polysaccharides (Figure 2). The band in the region of 1,654.62 cm^−1^ indicated the presence of OH flexural vibration of the polysaccharides. The band at around 1,452 cm^−1^ was assigned to CH (OCH_2_) flexural vibrations. The appearance of characteristic peak band around 1,049 cm^−1^ suggested the presence of CO stretching vibrations. The band around 929 and 815 cm^−1^ was assigned to the existence of fructose with β-type glycosidic bond. All the absorption bands listed above are FTIR spectroscopy characteristic peaks of garlic neutral polysaccharides.

### Changes in Saccharides During Acid Hydrolysis

It generates a large number of monosaccharides during the acid hydrolysis steps, which may influence the prebiotic effect because free sugar can be digested by humans and they have a high glycemic index (GI) as compared with oligosaccharides. Thus, the removal of fructose and glucose is also required to analyze their prebiotic properties.

Cation exchange chromatography and ultrafiltration are frequently used to purify the saccharides mixture (29). We used ultrafiltration to remove the monosacharides in our study, in which the *M*_W_ cut off (MWCO) of the membrane was 300 Da more than *M*_W_ of monosaccharide and less than *M*_W_ of disaccharide. As can be seen from Table 1, compared with GPs, the *M*_W_ of saccharides in GOs became smaller, suggesting that polysaccharides in GPs were degraded to oligosaccharides or monosaccharides *via* acid hydrolysis. The reducing sugar content after ultrafiltration was below 5%, and the content of oligosaccharides with average DP < 10 increased from 15 to 75%. The saccharides with different DP have different prebiotic effect. It found that the DP of 10 was an important physicochemical threshold. Inulin chains with a DP above 10 are fermented up to five times slower by colonic bacteria compared with inulin chains with a DP below 11 by *in vitro* fermentation experiments (8, 30).

**Table 1:** The molecular weight distribution of GPs and GOs.

**GPs**			**GOs**		
**M_n_**	**M_w_**	**Percentage/%**	**M_n_**	**M_w_**	**Percentage/%**
2,205	4,698	100	739	1,444	100
11,445	13,115	15.84	12,304	12,451	1.59
3,185	3,690	65.88	8,662	8,858	1.83
1,397	1,405	7.98	2,166	2,305	21.46
1,110	1,114	3.80	1,302	1,313	18.27
915	916	0.97	1,051	1,053	7.30
786	788	1.00	922	923	6.10
646	647	1.00	795	796	6.15
510	512	0.87	657	658	7.05
339	341	2.32	519	520	8.10
232	233	0.17	358	359	21.53
177	178	0.18	231	232	0.16
–	–	–	181	182	0.46

*M_n_, number-average molecular weight; M_w_, weight-average molecular weight*.

### Effect of Artificial Human Gastric Juice Hydrolysis

The prebiotic effect of non-digestibile oligosaccharides has been increasingly focused on (31). They can escape digestion in the upper gastrointestinal tract and reach the colon virtually intact. As a result, they can alter the colonic microflora by increasing numbers of saccharolytic species and reducing putrefactive microorganisms.

**Figure 3:**
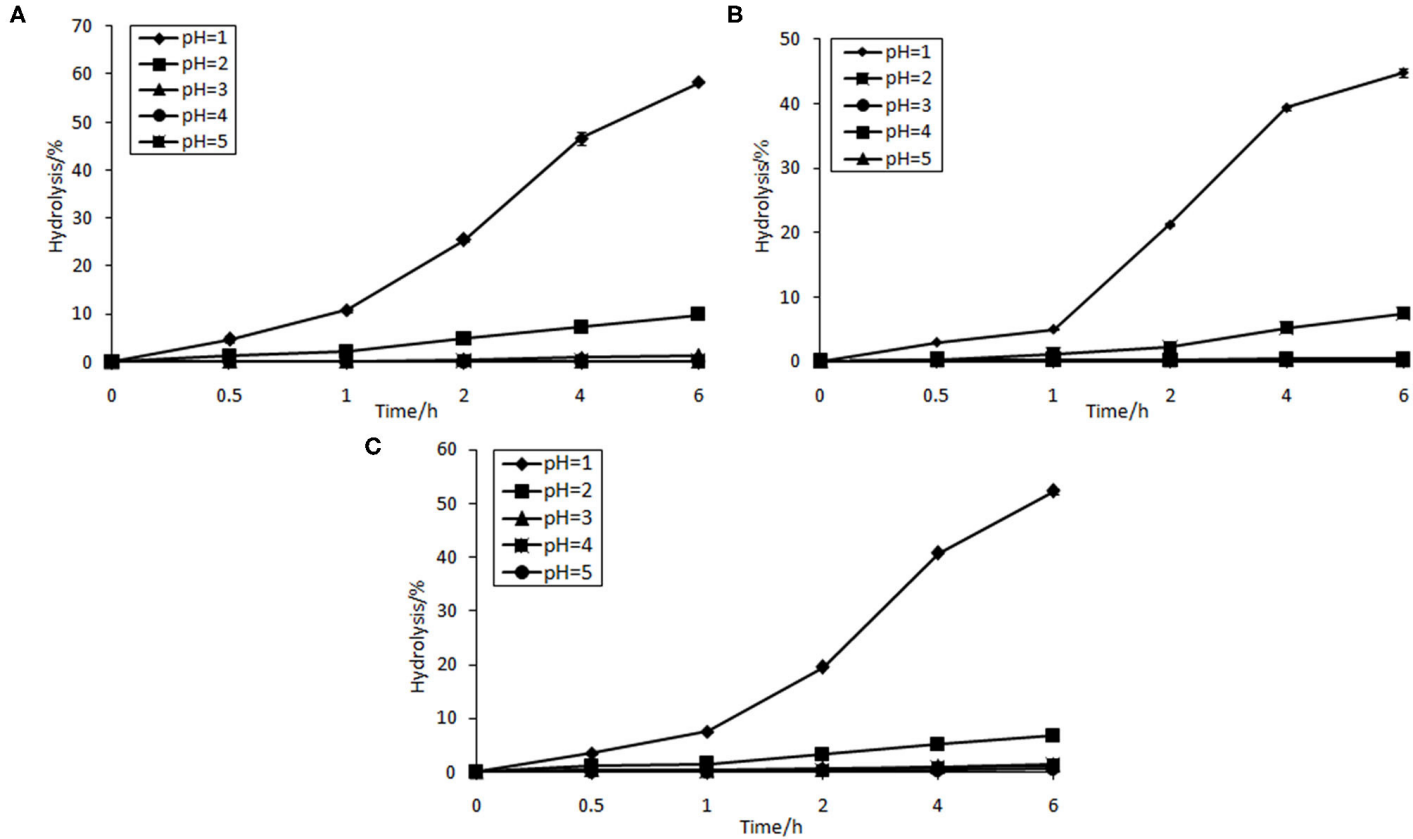
Resistance of FOS **(A)**, GPs **(B)**, and GOs **(C)** to various artificial gastric juice.

As can be seen from Figure 3, GPs and GOs were found to be resistant to artificial human gastric juice as compared with FOS. Percentage of hydrolysis increased with decreasing pH of artificial gastric juice. The hydrolytic degree of FOS at pH = 1 was 58.34%. However, that of GPs and GOs was 44.79 and 52.33%, respectively. The hydrolysis of FOS, GPs, and GOs was slow under the pH of 2–5, where the percentage of hydrolysis was <10%. However, food is usually retained in the human stomach for about 2 h (31). The results indicate that most of the GPs and GOs are not digested, and so they can reach the large intestine. The results gathered give a good indication that GPs and GOs can be regarded as potential prebiotic. Some other oligosaccharides were also studied, such as koji oligosaccharides and gluco-oligosaccharide produced by *Gluconobacter oxydans* NCIMB 4943, which had 100 and 98.4% resistance to artificial gastric acid, respectively (32). Moreover, dextran highly resists the acidic conditions in human digestive tract (33). Under acidic circumstance, the release rate of fructose during hydrolysis is dependent on the fructosyl chain ends concentration and average chain length distribution. The fructose production rate is expected to be much faster for the smaller oligomers, which have a relatively high content of fructosyl ends of chain (34).

### Effects of Different Saccharides on Prebiotic Growth

**Figure 4:**
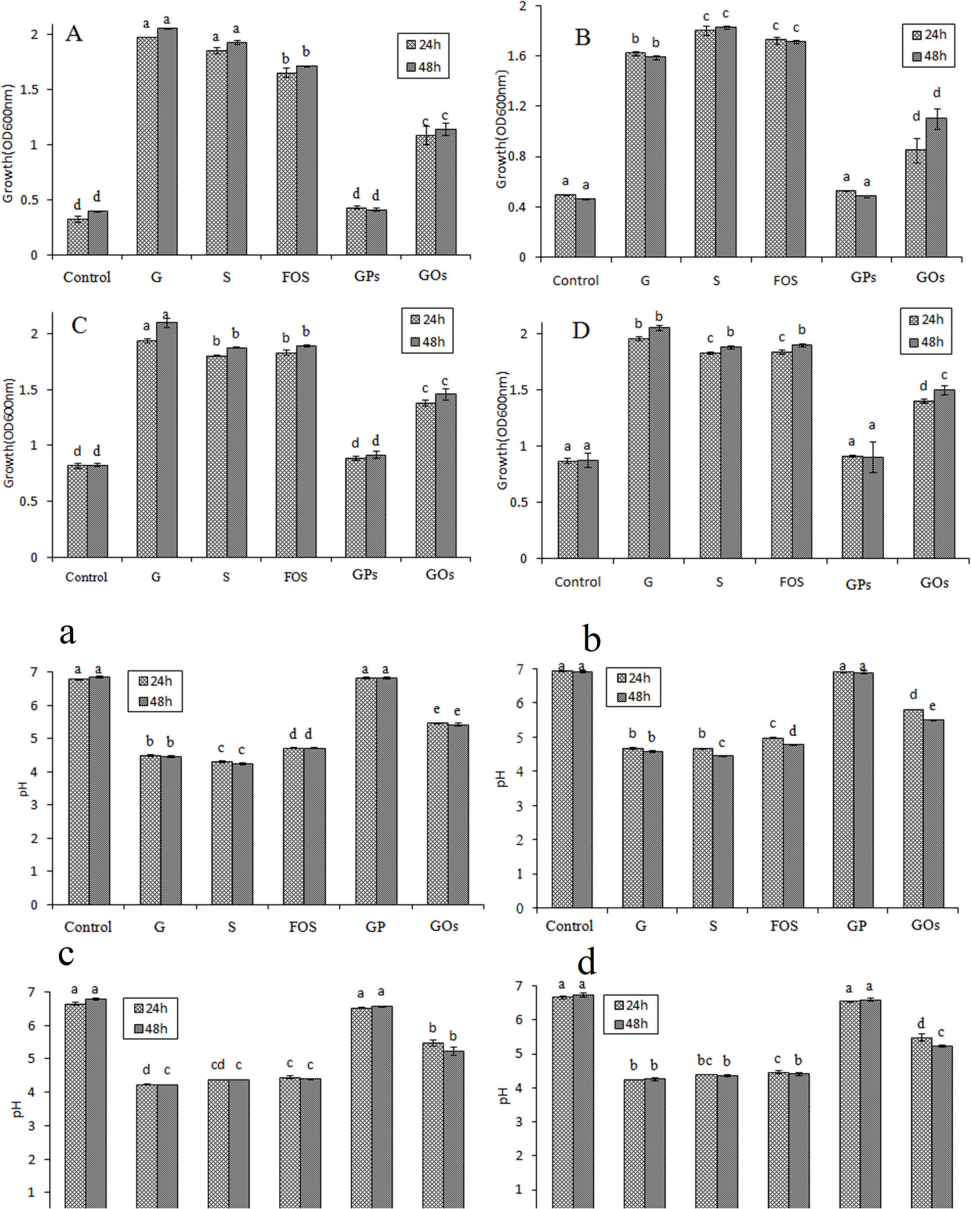
Effects on the growth of Lactobacillus and pH of the medium at 24 and 48 h in semisynthetic medium using glucose (G), sucrose (S), FOS, GPs, GOs as the sole carbon source and no carbon medium as control (A/a: *L. plantarum*; B/b: *L. johnsonii*; C/c: *L. paracasei*; D/d: *L. casei*.).

The OD value of lactobacillus means the growth of probiotics and effective utilization of different saccharides. As demonstrated in Figures 4A–D, all lactobacillus could utilize glucose, which was assigned as previously described (35). Different saccharides had different growth efficiency to them, and the OD value did not change significantly from 24 to 48 h, indicating that it reached a stable phase. The medium of control had a value, which meant these lactic acid bacteria could show a small amount of growth in no carbon medium. It was found that they could grow well in the medium containing glucose, sucrose, and FOS by OD value reaching to above 1.5, and GOs stimulated the growth by increasing its OD value, reaching above 1.0. However, GPs showed no significant difference compared with the control, and it had little effect on the growth of lactobacillus.

Glucose and sucrose are assimilated in the gastrointestinal tract; however, they cannot reach the colon. Though they promote the growth of beneficial bacteria, they are not considered as prebiotics. FOS were found to have the most significant effect on the growth of probiotics, however, GOs had a better effect on stimulating the growth of them than GPs, less than FOS. This may be attributable to this particular DP of different saccharides, which is a mixture of diverse DP fructans. It is found that the fructans with lower DP have a better prebiotic effect except disaccharide (14). The DP of saccharides between 3 and 4 has the best prebiotic effect, and when the DP > 5, the prebiotic effect decreases. The average DP of FOS is 3.2, which can significantly enhance the growth of lactobacillus. GPs mainly consists of polysaccharides with high *M*_W_ and a bit of oligofructose, resulting in the average DP to exceed more than 20. GOs is generated by GPs through acid hydrolysis. The content of high *M*_W_ polysaccharides decreased, but the oligofructose increased, which attributed to the decreasing of the average DP.

### Effects of Different Saccharides on the pH of Culture Mediums

The oligofructose can be decomposed into some kinds of acids in the growth process of probiotics while the pH of cultures will decrease. Therefore, a reduction in pH can be used as an indication of the prebiotic effect of the oligosaccharides incorporated in the culture broth (36). As shown in Figures 4a–d, the decrease of pH values in the cultures suggested that all the lactobacillus were able to utilize these saccharides. pH values of glucose, sucrose, and FOS decreased quickly to about 4.5 in 48 h; however, that of GOs was about 5.2 at the same time. The pH value of culture with GPs as carbon source showed little decrease, and there was no significant difference with that of the control. For quantitative comparison of the prebiotic effects, PI was calculated for the saccharides. PI represents a comparative prebiotic effect relationship with glucose. PI value of the medium at 48 h was shown in Table 2, and it was found that PI value of FOS wase above 0.75, indicating that FOS had significant effect on the growth of probiotic. PI value of GPs was nearly zero; however, that of GOs was above 0.5 except that in the culture of *L. plantarum* it was 0.45.

**Table 2:** PI value of the medium at 48 h.

**Bacterial strains**	**MRS-S**	**MRS-FOS**	**MRS-GPs**	**MRS-GOs**
*L. plantarum*	0.92 ± 0.020a	0.79 ± 0.017a	0.01 ± 0.003b	0.45 ± 0.060c
*L. johnsonii*	1.21 ± 0.010a	1.11 ± 0.009a	0.02 ± 0.008b	0.57 ± 0.075c
*L. paracasei*	0.83 ± 0.004a	0.84 ± 0.008a	0.07 ± 0.024b	0.50 ± 0.036c
*L. casei*	0.89 ± 0.012a	0.91 ± 0.013a	0.03 ± 0.120b	0.55 ± 0.037c

*The same line marked with the same letter indicates no significant difference (P > 0.05) and different letters indicate a significant difference (P < 0.05)*.

## Discussion

Acid thermal hydrolysis is traditionally used to obtain oligosaccharides and monosaccharides for several years. Usually, high temperatures combined with low acid concentrations result in the continuous hydrolysis of garlic polysaccharides, which was the end with fructose as the major single product and glucose as a minor hydrolysis product. The product is a process in which the average *M*_W_ distribution decreases with time. At the end of the reaction, all garlic polysaccharides transformed to fructose and a limited amount of glucose. So, oligosaccharides mixture with different *M*_W_s can be obtained at an appropriate time. In order to obtain these oligosaccharides and remove the monosaccharides, ultrafiltration system was used for purifying and concentrating them, and it was proved as an effective method. Inulin with lower DP is more active and shows higher degree of utilization (14). The garlic polysaccharides have the same results that GOs had, a better prebiotic effect than GPs, but less than FOS.

The biological activities had a close relationship to the chemical structure, composition, DP, linear or branched structure, as well as water solubility. Low DP of oligofructose is more rapid and more selectively fermented by lactobacilli than high DP of polysaccharides. This may be due to the fact that the low DP oligofructose means more non-reducing ends per unit mass, which favors attack by exo-acting enzymes (10). The population growth of the bacteria exerts a decreasing of pH (37). The bacteria degrade oligosaccharide and polysaccharide in two stages. In the first stage, they hydrolyze oligosaccharide into monomers by glycosidases. Then the monomers are metabolized to volatile fatty acids and gases, which attribute to the decreasing of pH (38). Moreover, pH decrease has a potentially important effect because these acids could reduce intestinal pH and restrict or prohibit the growth of many potential pathogens and putrefactive bacteria.

## Conclusion

In this study, the neutral polysaccharides from garlic were obtained, analyzed, acid hydrolyzed, and their prebiotic effect was then evaluated. The FTIR spectrum of GPs revealed the existence of fructose with a β-type glycosidic bond. The average *M*_w_ of GPs by GPC analysis was appeared to be 6.57 × 10^3^ Da. Monosaccharide composition by HPLC analysis showed that it was composed of fructose and glucose at a ratio of 4:1. After acid hydrolysis, a low *M*_W_ fraction including glucose and fructose of GOs was successfully removed to below 5% by ultrafiltration. The content of oligosaccharides with average DP < 10 increased from 15 to 75%. GPs and GOS had a stronger resistance to acid conditions in human stomach than FOS, and GOs showed better prebiotic properties on the growth of lactobacilli than GPs. Thus GOs are a potential source of prebiotics, which may be used as an ingredient in functional food and nutraceutical products.

## Data Availability Statement

The original contributions presented in the study are included in the article/Supplementary Material, further inquiries can be directed to the corresponding author/s.

## Author Contributions

XL: investigation, software, and writing an original draft. NL: conceptualization, methodology, writing—review and editing, and supervision. RZ: supervision, project administration, and data curation. MZ: project administration, data curation. XC: manuscript revision. YX: manuscript revision. XQ: methodology, supervision. All the authors reviewed and accepted the content of the final manuscript.

## Funding

This study was supported by the National Natural Science Foundation of China (31801559), Shandong Agricultural Applied Technology Innovation Project (2018), Major Scientific and Technological Innovation Project of Shandong Province (2019JZZY020607).

## Conflict of Interest

The authors declare that the research was conducted in the absence of any commercial or financial relationships that could be construed as a potential conflict of interest.

## Publisher's Note

All claims expressed in this article are solely those of the authors and do not necessarily represent those of their affiliated organizations, or those of the publisher, the editors and the reviewers. Any product that may be evaluated in this article, or claim that may be made by its manufacturer, is not guaranteed or endorsed by the publisher.
